# Addition of Mitoquinone (MitoQ) to Fresh Human Sperm Enhances Sperm Motility without Attenuating Viability

**DOI:** 10.3390/biology13090653

**Published:** 2024-08-23

**Authors:** Nehad Al-Tarayra, Zina M. Al-Alami, Abdelkader Battah, Nadia Muhaidat

**Affiliations:** 1 Department of Pathology, Microbiology and Forensic Medicine, School of Medicine, The University of Jordan, Amman 11942, Jordan; nehadh.tarayra95@gmail.com (N.A.-T.); akbattah@ju.edu.jo (A.B.); 2 Fertility Center, Istishari Hospital, 44 Kindi Street, Amman 11954, Jordan; 3 Department of Basic Medical Sciences, Faculty of Allied Medical Sciences, Al-Ahliyya Amman University, Amman 19328, Jordan; z.alalami@ammanu.edu.jo; 4 Department of Obstetrics and Gynaecology, School of Medicine, The University of Jordan, Amman 11942, Jordan

**Keywords:** antioxidant, human sperm, MitoQ, Mitoquinone, sperm motility, sperm viability, semen fluid analysis, sustainable healthcare

## Abstract

**Simple Summary:**

When a couple faces infertility challenges like sperm motility issues, they often visit fertility clinics for assistance. Poor sperm motility can delay fertilization, making natural conception difficult. In an andrology laboratory, embryologists test the semen sample. Before assisted reproduction procedures, embryologists usually perform a group of steps to prepare the semen sample and to isolate the best sperms. These technical steps might harm the sperms, because they become susceptible to something so called; oxidative stress, which occurs when too many harmful molecules called free radicals accumulate in the test tube, leading to cell damage and death. A group of chemicals called antioxidants can neutralize these free radicals. The researchers in this study investigated the therapeutic effects of Mitoquinone, which is a mitochondria-targeted antioxidant, on the motility of human sperms exposed to oxidative stress during in vitro procedures. Mitoquinone as an antioxidant might have therapeutic potential for sperms’ oxidative-damage-associated disorders. This investigation paves the route for future research to confirm the beneficial role of Mitoquinone supplementation in improving and enhancing sperm quality during in vitro processes in the andrology laboratories.

**Abstract:**

The preparation of human sperm in an andrology laboratory subjects it to oxidative stress. Reactive oxygen species are produced by mitochondria, making it susceptible to oxidative damage; hence, mitochondria-targeted antioxidants like Mitoquinone (MitoQ) might have therapeutic potential for oxidative-damage-associated disorders. The current research aims to establish whether MitoQ has any positive effects during in vitro preparation of fresh human sperm. Viability and motility are evaluated to determine the effective MitoQ concentration and to assess whether MitoQ supplementation is affected by sperm concentration by incubating normospermia semen samples at 37 °C for 2 h and 4 h, respectively. The effect of semen centrifugation following supplementation of 20 × 10^6^ sperm/mL with 200 nM MitoQ is also assessed by measuring viability, motility and sperm DNA fragmentation. The best sperm motility is achieved after 2 h of incubation with 200 nM MitoQ at 37 °C. Sperm concentration of 20 × 10^6^ sperm/mL is the best concentration where 200 nM MitoQ works efficiently. For semen centrifugation at 300× *g* for 20 min, supplementation with 200 nM MitoQ shows higher sperm motility. The current results demonstrate that MitoQ supplementation during in vitro human semen preparation procedures positively affects fresh sperm motility without affecting viability or increasing DNA fragmentation.

## 1. Introduction

The balance between producing and scavenging reactive oxygen species (ROS) plays a crucial role in the ability of the sperm to fertilize the oocyte, since high concentrations of ROS cause sperm defects, including viability, motility and fertilization ability, while controlled concentrations of ROS might be helpful in sperm functioning [1].

In eukaryotic cells, ROS are produced by the mitochondria, making them susceptible to oxidative damage; hence, mitochondria-targeted antioxidants are widely thought to have therapeutic potential for oxidative-damage-associated disorders [2].

Triphenylphosphonium (TPP) is an example of a lipophilic cation that can be used as a vehicle to transport antioxidants to mitochondria. It is commonly used in the development of mitochondria-targeted antioxidants, such as Mitoquinone (MitoQ), which is a derivative of ubiquinone (Coenzyme Q), which is covalently targeted to mitochondria via attachment to TTP. MitoQ is used in numerous animal and human studies and human clinical trials, including fertility and andrology studies [2,3,4].

A limited number of articles addressing frozen–thawed and cryopreserved human and non-human sperm samples were published to determine the influence of MitoQ on sperm. One of fhe first reports published on this topic was in 2014, addressing the addition of MitoQ to the freezing extender, where it was documented that MitoQ addition increased the post-thaw viability while decreasing ROS production and lipid peroxidation in yellow catfish [5]. Lately, Moradi Gardeshi and his group concluded that adding MitoQ to the freezing medium provided advantages for sperm cryopreservation, as it improved human sperm functional parameters, particularly the total and progressive motility and sperm viability [6]. This confirms previous publications on the subject of MitoQ improvement of human sperm motility during cryopreservation [7,8].

A few papers on MitoQ’s effect on the cryopreservation of cattle sperm have also been published; supplementation of MitoQ to the semen extender could efficiently improve the quality and fertility rate of cryopreserved sheep and goat semen and could be an additive for supplementation of the semen extender to improve the quality of cryopreserved sperm [9,10,11,12]. Other articles showed that MitoQ could be a promising additive for supplementation of rooster semen cryopreservation extender to enhance rooster sperm parameters after freezing and thawing [13,14,15]. Other studies documented that MitoQ could relieve oxidative stress (OS) and/or improve boar and buffalo bull sperm functions, regulate sperm antioxidant capacity and increase the post-thaw progressive motility and/or viability [16,17,18].

During assisted reproductive technology (ART), human sperm becomes susceptible to a wide range of in vitro procedures, which subject it to many sources of OS, either endogenously from the sperm itself or exogenously from the manipulation procedures, such as visible light exposure, culture media, cryopreservation, oxygen tension, pH, temperature and centrifugation [19]. Centrifugation is one of the extensively used procedures in the andrology laboratory, particularly during sperm selection procedures, such as swim-up techniques and density gradients. Centrifugation forces applied on sperm result in high production of ROS [20]. Therefore, the suggestion that antioxidants could be added as supplements to the culture media during sperm preparation techniques is promising, necessary and vital. Since sperm motility in post-wash semen samples that is above the lowest limit recommended by the WHO is considered an important factor in intrauterine sperm injection (IUI) outcomes [21], the current study concentrated on finding a supplement that can increase total motility, with the intention to help men with poor total motility reach the lower total motility threshold needed for successful IUI.

To the best of the authors’ knowledge, the addition of MitoQ as an antioxidant to the preparation media during in vitro preparation of semen samples was not studied previously. The objective of this study was to investigate the therapeutic effects of MitoQ, the mitochondria-targeted antioxidant, on the motility of human sperm exposed to oxidative stress during in vitro procedures. Specifically, the study aimed to determine MitoQ optimal concentration and sperm concentration that would improve sperm motility while maintaining sperm viability and not affecting DNA fragmentation. Through this investigation, the study sought to provide proof confirming the beneficial role of MitoQ supplementation in improving and enhancing sperm quality during andrology laboratory processes like swim-up selection techniques before IUI and in vitro fertilization (IVF).

## 2. Materials and Methods

### 2.1. Ethical Approval and Consent Form

The experiments were conducted according to global ethical guidelines under the Institutional Review Board (IRB) approval number IRBIH/NHA/17.8.23 granted by the IRB/IEC committee at Istishari Hospital, Amman-Jordan, on 17 August 2023. All patients voluntarily signed an informed consent form before participating in the study after being provided with an explanation regarding the aim and procedures of the experiments.

### 2.2. Participants and Semen Sample Processing

Previous clinical studies on human sperm [22] and similar in vitro studies on human sperm recruited patient samples of fifteen [23], twenty [6], thirty-four [7] and sixty [8] normospermic patients or others. In total, sixty-seven normozoospermic males participated in the current study. They were chosen and examined based on normal semen fluid analysis in accordance with the recommendations set out by the World Health Organization (2021) [24]. Samples were collected via masturbation after a 3–5-day abstinence period. Semen samples were assessed in terms of volume, sperm concentration, total cell count, motility and morphology after being liquefied at 37 °C for 30 min. Sperm motility was read using a Mackler chamber, and nigrosin–eosin staining was used to measure sperm viability. For sperm motility and viability, duplicate and triplicate readings of 200 sperms were examined, respectively; the results were reported as a percentage.

### 2.3. Preparation of MitoQ Solution

To prepare the concentrations of MitoQ (GLPBIO, Montclair, CA, USA), it was solubilized in dimethyl sulfoxide (DMSO) (Santa Cruz Biotechnology, Dallas, TX, USA) according to the manufacturer’s instructions.

### 2.4. Determination of the Most Effective MitoQ Concentration

To determine the most effective MitoQ concentration, 16 semen samples from the 67 recruited participants were prepared to a final concentration of 20 × 10^6^ sperm/mL. Each sample was divided into six tubes. The six tubes of each sample were supplemented with the following concentrations of MitoQ: 50, 100, 150, 200 and 400 nM, and phosphate-buffered saline (PBS) was added to the sixth tube to represent 0 nM MitoQ. Sperm motility and viability were assessed after incubation at 37 °C for 2 and 4 h.

### 2.5. Determining the Best Sperm Concentration That Is Most Efficiently Enhanced by MitoQ

To determine whether MitoQ could be affected by sperm concentration and to pinpoint the sperm concentration that is most efficiently enhanced by MitoQ, semen samples from 12 out of the 67 recruited participants were prepared to a final concentration of 5, 10, 20 and 40 *×* 10^6^ sperm/mL. Every concentration from every sample was divided into two tubes: one was supplemented with 200 nM MitoQ and the other with PBS as a control. After 2 h of incubation at 37 °C, the motility and viability of the sperm were measured.

### 2.6. Impact of MitoQ on Semen Centrifugation and DNA Fragmentation

Based on the results of the previous experiment, 200 nM was deemed to be the most effective MitoQ concentration, and 20 × 10^6^ sperm/mL was the best sperm concentration. To determine whether there were any protective effects of MitoQ after centrifugation, 19 semen samples from the 67 recruited participants were prepared to a final concentration of 20 × 10^6^ sperm/mL. Each sample was divided into two tubes: one was supplemented with 200 nM MitoQ and the other with PBS as a control. Following centrifugation at 300× *g* for 20 min, sperm viability and motility were assessed.

To assess the effect of MitoQ on DNA fragmentation, 20 normozoospermia donor samples from the 67 recruited participants were utilized. Every sample was prepared to a final concentration of 20 × 10^6^ sperm/mL, then divided into two tubes: one was supplemented with 200 nM MitoQ and the other with PBS as a control. Ten samples were incubated for 2 h at 37 °C, and the other ten were centrifuged at 300× *g* for 20 min. Sperm DNA fragmentation was measured as a percentage of fragmented sperm divided by total sperm using Halosperm DNA fragmentation kit (Halotech, Madrid, Spain).

### 2.7. Statistical Analysis

A statistical analysis was conducted using SPSS software version 25. Descriptive data were reported as means and standard deviations. All inferential statistics, including independent sample *t*-tests, were used at a significance level of *p* < 0.05 between the interventional and control groups. The data were normally distributed using the Shapiro–Wilk test, and the homogeneity of variance was assessed using the Levene test.

## 3. Results

### 3.1. The Most Effective MitoQ Concentration

**Figure 1:**
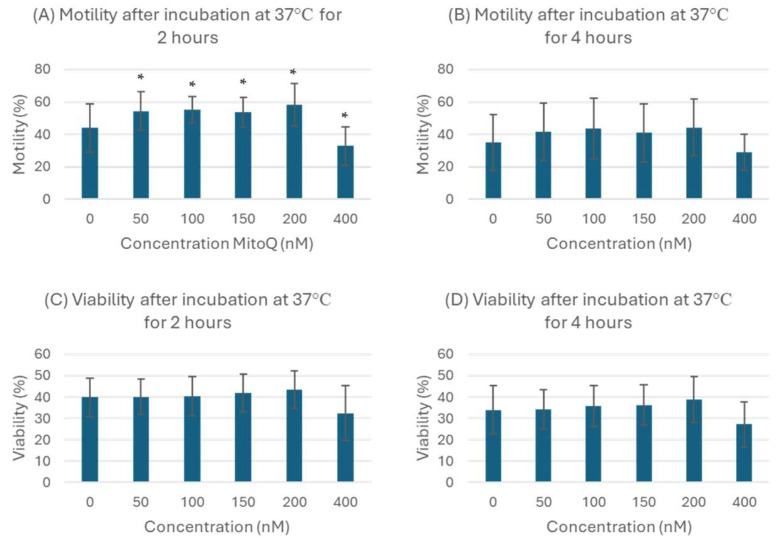
Effect of different concentrations of MitoQ on sperm motility (**A**,**B**) and viability (**C**,**D**) after incubation at 37 °C for 2 h and 4 h. Data are presented as mean ± standard deviation, n = 16. * indicates that the *p* value is < 0.05, which means that the values are significantly different.

After the incubation of sixteen semen samples for 2 h and 4 h at 37 °C, the effects of various MitoQ concentrations (50, 100, 150, 200 and 400 nM) on sperm motility and viability were examined (Figure 1). After 2 h of incubation, sperm motility increased significantly upon MitoQ supplementation at 50, 100, 150 and 200 nM (Figure 1A) (54.4 ± 11.8, 55.3 ± 8.1, 53.7 ± 8.9, 58.3 ± 13.2%, respectively) compared with control (44.0 ± 14.9; *p* < 0.05). The concentration of 200 nM showed the highest increase in motility (58.3 ± 13.2%) after 2 h of incubation. However, incubating the sperm for 2 h with 400 nM MitoQ significantly reduced the motility (32.8 ± 11.9%).

Incubation for 4 h at 37 °C with 50, 100, 150, 200 and 400 nM did not affect sperm motility either positively or negatively (Figure 1B). Furthermore, none of the used MitoQ concentrations affected sperm viability after incubation with MitoQ concentrations either for 2 h or for 4 h (Figure 1C,D). Therefore, the best sperm motility was achieved after sperm incubation with 200 nM MitoQ for 2 h.

### 3.2. The Sperm Concentration That Is Most Efficiently Enhanced by MitoQ

**Figure 2:**
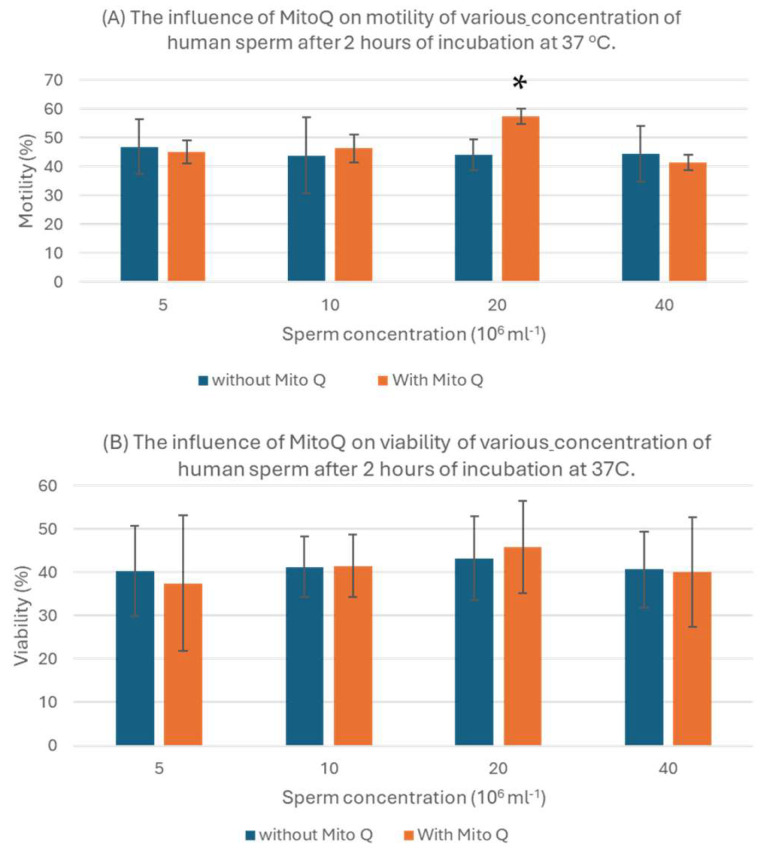
Determining the sperm concentration that is most efficiently enhanced by MitoQ, as reflected by sperm motility (**A**) and sperm viability (**B**) with and without 200 nM MitoQ. n = 12. Data are represented as mean ± standard deviation. * indicates that the *p* value is < 0.05, which means that the values are significantly different.

Semen samples from twelve donors were prepared to a final concentration of 5, 10, 20 and 40 × 10^6^ sperm/mL. Every concentration from every sample was divided into two tubes, supplemented with either 200 nM MitoQ or PBS. After 2 h of incubation at 37 °C, the motility and viability of the sperm were measured (Figure 2). Sperm concentration 20 × 10^6^ sperm/mL was the sperm concentration in which sperm motility was most efficiently enhanced in samples supplemented with 200 nM MitoQ (*p* < 0.05). The motility of sperms in the other sperm concentrations (5, 10, 40 × 10^6^ sperm/mL) was not enhanced by supplementation of 200 nM MitoQ (Figure 2A). None of the sperm concentrations (5, 10, 20, 40 × 10^6^ sperm/mL) affected the viability of sperms after incubation with 200 nM MitoQ (Figure 2B).

### 3.3. Impact of MitoQ on Semen Centrifugation

**Figure 3:**
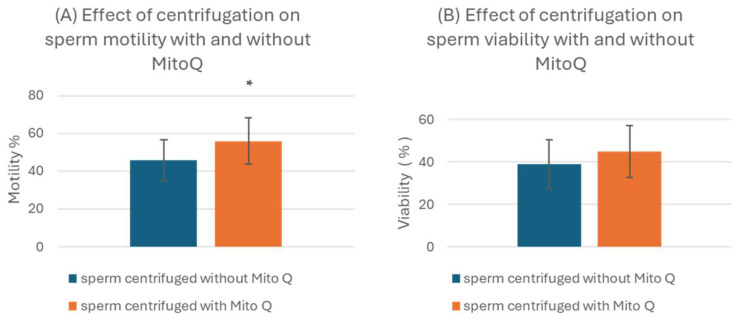
Effect of centrifugation for 20 min at 300× *g* on (**A**) sperm motility and (**B**) sperm viability with and without 200 nM MitoQ. n = 19. All data are mean ± standard deviation. * indicates that the *p* value is < 0.05, which means that the values are significantly different.

Following centrifugation at 300× *g* for 20 min, sperm motility and viability were assessed in both aliquots: one supplemented with 200 nM MitoQ and the control tube with PBS (Figure 3). Sperms centrifuged with 200 nM MitoQ showed significantly higher average sperm motility (55.9 ± 12.2) than the control group (45.7 ± 11.0) (*p* < 0.05) (Figure 3A). Nevertheless, 200 nM MitoQ did not affect sperm viability (44.8 ± 12.1) compared to the control group (38.9 ± 11.4) (Figure 3B).

### 3.4. Impact of MitoQ on Sperm DNA Fragmentation

**Figure 4:**
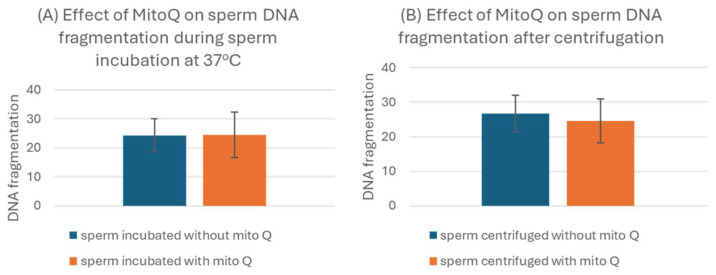
Effect of MitoQ on sperm DNA fragmentation during (**A**) sperm incubation at 37 °C for 2 h with 200 nM (n = 10) and (**B**) centrifugation in the presence of 200 nM MitoQ for 20 min at 300× *g* (n = 10). *p* > 0.05.

To assess the effect of MitoQ on DNA fragmentation, ten semen samples were incubated for 2 h at 37 °C with and without MitoQ, and the other ten were centrifuged at 300× *g* for 20 min with and without MitoQ. The concentration of 200 nM MitoQ did not affect DNA fragmentation either negatively or positively and either after incubation or after centrifugation (Figure 4).

## 4. Discussion

In the andrology laboratories, during the preparation procedures of ART, fresh human sperm is exposed to many sources of OS; one of the major sources is centrifugation during sperm selection procedures, such as swim-up techniques and density gradients [20]. Thus, suggesting novel antioxidants as additives to the culture media throughout the sperm preparation process has become essential. The current work studied the antioxidant MitoQ as a potential supplement, and it aimed to figure out whether MitoQ could have any positive effects during the preparation of fresh sperm in the andrology laboratory.

The current results demonstrated that supplementation of MitoQ positively affected fresh sperm motility, during which sperm viability and DNA fragmentation were not affected either positively or negatively.

The concentration of 200 nM MitoQ resulted in the best sperm motility, both when the fresh sperm sample was incubated for 2 h at 37 °C—as this concentration resulted in a 14.3% increase in total motility compared with the fresh control group—and when it was added to the sample during centrifugation at 300× *g* for 20 min. Interestingly, incubation of fresh human sperm for only 2 h at 37 °C with 50, 100 and 150 nM MitoQ also significantly increased its motility, but 200 nM MitoQ showed the highest increase in motility, whereas 400 nM adversely reduced sperm motility. Additionally, the current results showed that the concentration of 200 nM MitoQ worked best with a sperm concentration of 20 × 10^6^ sperm/mL to enhance sperm motility after 2 h incubation at 37 °C and after centrifugation at 300× *g* for 20 min.

Based on the available evidence and the literature that the authors could access, the effect of MitoQ on fresh human sperm motility was not studied previously. Accordingly, the current findings were compared with the results of the research groups who worked on its effect following cryopreservation and those who worked on non-human samples.

In two articles published to assess the effect of MitoQ on restoring human sperm quality after cryopreservation, one group reported that adding 500 nM MitoQ to the cryopreservation media could increase the total motility by 15.5% more than the frozen control [7]. Meanwhile, the other group reported that total sperm motility significantly increased in the 2 nM MitoQ group [6]. Both articles were consistent with our results, since MitoQ increased the total motility. Nevertheless, both groups worked with MitoQ as an additive to the cryopreservation media.

Furthermore, the current results are consistent with articles that suggested that MitoQ could be a promising additive for supplementation of rooster semen cryopreservation extender to enhance rooster sperm parameters after freezing and thawing. Nevertheless, the effective concentrations varied between 10 and 100 nM [13] or 50 and 100 pMol [14] and 150 nM [15].

Other studies were conducted on bull semen; one of the reports revealed that the addition of 0, 0.2, 2 and 20 nM MitoQ to the semen extender before cryopreservation of bull sperm could not improve bull sperm quality after thawing, and surprisingly, 20 nM MitoQ increased ROS synthesis in the frozen–thawed sperm. Nevertheless, they did not record any negative effects on the other sperm parameters [25]. On the contrary, MitoQ (0.02 μM) could relieve OS by regulating mitochondrial functionality in buffalo bull sperm during cryopreservation [16], and adding 10 μM MitoQ resulted in the highest boar sperm motility and sperm quality after thawing. Nevertheless, excessive MitoQ (40 μM) supplementation induced a reduction in sperm motility parameters, sperm quality and also antioxidant status of boar semen [17]. Moreover, the study of Tiwari et al., documented that MitoQ (200 nM) improved in vitro sperm functions of cryopreserved buffalo (*Bubalus bubalis*) semen and increased post-thaw progressive motility and viability [18].

Moreover, 10 and 100 nM MitoQ resulted in higher total motility in ram sperm [10]. In addition, the concentrations of 10 and 100 nM MitoQ were able to increase the motility of post-thawed sperm in sheep, suggesting that they could be an effective supplement to be added to the semen of rams during the cryopreservation programs [9]. Moreover, the addition of 150 nM MitoQ to the extender enhanced the motility of cryopreserved Hu sheep [12]. Their results were consistent with ours, since 100 and 150 nM MitoQ increased the motility significantly.

These differences in the concentrations could be caused by the difference in the species or because the research groups worked on cryopreserved samples, not on fresh semen samples; in particular, cryopreservation could result in a reduction in the quality and function of sperm through disturbance of the function and ultrastructure of the mitochondria, resulting in lowering the energy and increasing the OS [26].

It is worth mentioning here that the current results are consistent with what was reported by Liu and his group when they investigated the role of MitoQ in post-thawed human sperm; they documented that the percentage of the total rate of sperm motility was significantly higher following supplementation with 200 nmol/L MitoQ [8]. This was also confirmed by the results of Kumar and his group, who documented that MitoQ protected human sperm motility after vitrification [27].

The current study has some limitations, the first being the sample size. In Jordan, there are no sperm banks due to religious and cultural issues; the research on sperm relies only on patients/participants who visit the clinics and agree to volunteer and to sign the consent forms. Nevertheless, the sample size used in this study agrees with the sample sizes used in many similar previously published studies. Moreover, this study is an ex vivo pre-clinical study, which is considered a representative example that can help generate data for power calculations for larger studies.

Moreover, our main objective was to find an antioxidant that enhances sperm motility for future use in larger scale clinical studies that focus on sperm preparation for IVF and IUI without affecting the viability and without causing DNA fragmentation. Our aim was not to perform semen fluid analysis or to find an antioxidant that improves sperm count or morphology, especially considering that the study was conducted among normospermia participants. Nevertheless, assessing only motility and viability might not be sufficient, and future work must include other sperm parameters. In addition, further studies on sperm mitochondria, their redox oxidant levels, as well as the molecules affecting sperm quality should be conducted to support the current results.

Some future work is also suggested. The current study only investigated the following MitoQ concentrations: 50, 100, 150, 200 and 400 nM. It was noted that 200 nM was the best concentration that increased fresh sperm motility upon incubation, whereas 400 nM showed a decline in sperm motility under the same circumstances. It is suggested and recommended that future work should include new concentrations that range between 200 and 400 nM to find the most effective concentration. Moreover, samples were only centrifuged at 300× *g* for 20 min. It is therefore also recommended to centrifuge samples with different forces and times to determine the best centrifugation force and time that have the best positive effects on fresh sperm.

## 5. Conclusions

In conclusion, 200 nM MitoQ seems to be effective in enhancing and increasing human sperm motility during in vitro sperm preparation procedures, particularly if the fresh sperm sample is prepared at 20 × 10^6^ sperm/mL and if the sample is incubated at 37 °C with 200 nM MitoQ for 2 h. Through this investigation, the study paves the route for future research to confirm the beneficial role of MitoQ supplementation in improving and enhancing sperm quality during in vitro laboratory processes in the andrology laboratories.

## Data Availability

Data analyzed or generated during this study are included in this manuscript.
